# “Looking for a *Golden* Needle in the Haystack”: Perspectives on Talent Identification and Development in Paralympic Sport

**DOI:** 10.3389/fspor.2021.635977

**Published:** 2021-04-08

**Authors:** Nima Dehghansai, Ross A. Pinder, Joe Baker

**Affiliations:** ^1^School of Kinesiology and Health Science, York University, Toronto, ON, Canada; ^2^Paralympic Innovation, Paralympics Australia, Adelaide, SA, Australia

**Keywords:** athlete development, athlete selection, sport classification, recruitment strategies, disability sport, athletes with impairments, para sport, female athletes

## Abstract

Despite rapid increases in research on talent identification and development in able-bodied sports, there remains limited knowledge regarding how talent is identified and developed in Paralympic contexts. The purpose of this study was to capture the perspectives of experts (coaches, high-performance managers, and pathway specialists) working in elite Paralympic sport to better understand how they conceptualize, measure, and develop talent. Eight coaches and three performance directors from six Paralympic sports, along with two pathway specialists from Paralympics Australia participated in semi-structured interviews. The results suggest impairment type and, therefore, classification are key indicators of identification and anticipated success, highlighting the importance of educating talent selectors in these areas. In addition, familial (e.g., overprotectiveness, sporting background) and biopsychosocial factors (e.g., resilience, work-ethic, sport-specific skills, other life commitments) were noted as being influential when selecting athletes. There were concerns regarding the disproportionately low number of female athletes in the system, suggesting a need for new initiatives to support early-entry points for female athletes (e.g., education on the benefits of sport participation, supportive environments). High-performance staff also lacked resources to better understand the nuances associated with different impairments and their implications (physiological response to training, associated psychological stresses from injury, identity change). Recruitment strategies included “talent search” days, collaborations with school programs and rehabilitation centers, and helping local clubs support “drop-in” athletes. However, limited funding impacted the sustainability of programs, resulting in a regular turnover of staff, loss of intellectual property, and a weakened pathway system. Results from this study generated several practical implications and future directions for research.

## Introduction

Identifying early-career performance indicators that predict future performance in high-performance sport has been difficult (Schorer et al., 2017; Johnston et al., 2018; Johnston and Baker, 2020). This has led to increased attention to understanding the meaning of “talent” and its indicators, as well as the optimal environments to nurture and maximize athletes' potential (Abbott and Collins, 2004; Baker et al., 2018). From an identification perspective, coaches and scouts assess and select primarily based on physical and physiological attributes (Baker et al., 2020) using a blend of techniques and protocols including intuition (Christensen, 2015; Lund and Söderström, 2017) and testing batteries (Gabbett, 2009). From a developmental perspective, there is a wide range of variables that influence athletes' trajectories including demographic (e.g., socioeconomic status, family status, sporting background, Côté, 1999; Post et al., 2018), physical and psychological (e.g., resilience, growth and maturation, Baxter-Jones et al., 2002; Roberts et al., 2019), sporting (e.g., quality coaching, coach-athlete relationship, Erickson and Côté, 2016), social (e.g., peers, Ullrich-French and Smith, 2009), environmental (e.g., access to facilities, Estabrooks et al., 2003), and political factors (e.g., policy for access to sport, funding, Barker-Ruchti et al., 2018). This breadth of research has informed stakeholders (i.e., sport organizations, coaches, athletes, families) of key factors that can impact the identification and development of an athlete. While, to a degree, there have been some improvements in our understanding of talent (Tetlock, 2016), this work has predominantly been done in able-bodied (AB) settings with very limited information on the processes in Paralympic (or other disability sport) contexts.

On the one hand, there may be some crossovers between AB and Paralympic contexts, such as the importance of family support and the quality of coach-athlete relationships. On the other hand, however, disability-related factors (impairment^1^ type, timing and nature of the impairment, potential classification) introduce levels of complexity that impact the range of factors that may be considered during athlete identification and development. For example, classification systems in Paralympic sports are designed to minimize the impact of impairment on the outcome (e.g., athlete's performance) (International Paralympic Committee, (n.d.)). In the limited research in this context, researchers have shown athletes' impairment and their potential classification to be a key indicator to the identification and potential success in their sport (Radtke and Doll-Tepper, 2014; Patatas et al., 2020).

Athletes in Paralympic sport enter sports at different ages based on the timing of impairment, thereby influencing how coaches consider athletes' readiness based on both their training and chronological ages (Radtke and Doll-Tepper, 2014).

Work by Dehghansai et al. (2017a, 2020b,c,d,f), Dehghansai and Baker (2020), and Lemez et al. (2020) highlighted the variability of development amongst Paralympic sport athletes and the need to consider the dynamic and complex interaction of factors influencing athletes' development from a holistic lens.

It is difficult to inform policy and structure optimal developmental environments with limited empirical evidence (Dehghansai et al., 2017b). Existing literature in this area has highlighted a need for organizations to optimize limited funding and support through the upskilling of (typically volunteer) development coaches with limited knowledge of disability, enhance cooperation between rehabilitation centers and school systems as these environments are entry points for athletes with impairments, and emphasize the importance of personal, financial, and infrastructural resources to support the identification and development process (Radtke and Doll-Tepper, 2014; Mann et al., 2017; Patatas et al., 2020).

In order to better understand the key components associated with talent identification and development, it is imperative to recognize the experience of the key stakeholders involved directly in the identification^2^ of athletes (i.e., coaches, high-performance managers, talent specialists). The purpose of this study was to extend this limited research base through a focused examination of the Australian Paralympic system's approach to talent identification and development. More specifically, we aimed to better understand current approaches, challenges, and strategies utilized by coaches, high-performance managers, and pathway specialists.

## Method

### Participants

#### Recruitment

High-performance (HP) staff from Paralympics Australia and Australian Paralympic sports were contacted by the lead author. Eight coaches and three performance directors from six different Paralympic sports (boccia, Para athletics, Para cycling, Para table-tennis, wheelchair tennis, wheelchair rugby), along with two pathway specialists from Paralympics Australia agreed to participate in this study. Interviews were held in person and based on the responses, the authors felt data saturation was reached and no further recruitment was required. Interviews ranged between 52 and 114 min in duration. Participants were assigned pseudonyms to protect their identity.

#### Background

Twelve out of 13 participants were ex-athletes (AB or Paralympic) and athletic successes ranged from the national level to Paralympic medalist. All reported being in their current role for longer than a full Paralympic cycle (i.e., were involved at the previous Rio 2016 Paralympic Games), and they either attended a Paralympic Games as a coach or coached an athlete who competed at the Games (i.e., they were the private coach that trained the athlete, but did not attend with the athlete due to limited accreditations). Ten out of 13 reported having an athlete medal at the Paralympic Games directly under their guidance. Three participants reported having an impairment themselves, while another two were trained classifiers.

### Methodology

Semi-structured interviews were used to capture participants' experiences, challenges, and perspectives of talent identification and development in the Paralympic context. The open-ended interviews strengthened the quality of data by eliciting meaningful conversations allowing participants to express their experiences.

#### Philosophical Assumption

This study was grounded ontologically and epistemologically in critical realism (CR, i.e., a reality exists which is experienced by individuals through a world that is constructed by social discourse, Fletcher, 2017). The CR approach allowed the search for underlying causal relationships in a world that is subjective and often unmeasurable. Thus, understanding multiple perspectives, the tendencies and meanings were drawn from participants' experiences (Creswell, 2014; Smith, 2015; Cooper and Ewing, 2019), and discerning key elements across different Paralympic environments in various roles allowed depiction of the larger structure of the “talent” narrative (Wiggins and Potter, 2008; Smith et al., 2016). Therefore, recognizing these narratives helped understand the larger system and can guide strategic directives to improve talent identification and development across Paralympic sport in Australia.

#### Methodological Rigor

A guiding list of criteria was set out to establish the rigor of this study design (Tracy, 2010; Sparkes and Smith, 2014; Smith and McGannon, 2017). While talent identification and development has been studied in the AB context, there remains a void in the Paralympic context which leaves stakeholders with very limited empirical evidence to inform their decisions, demonstrating the *worthiness of topic, significant contribution*, and *practicality*. Our methods align with previous literature (Cooper and Ewing, 2019; Dehghansai et al., 2019), using theoretical constructs, data collection, and analysis processes that have been accepted and regularly employed in sports research, highlighting the *rich rigor. Credibility* was achieved through the multivocality of participants, across numerous sports in a wide range of roles, enabling the view of talent in the Paralympic context from multiple lenses. *Ethical* considerations were taken to retain participant anonymity in this small community. We also considered the working relationship between a member of the research team (affiliated with Paralympics Australia) and the participants (i.e., *relational ethics*, Bergum and Dossetor, 2005). The lead author, with no previous working relationship, led the interviews and reiterated to participants that their contributions would be kept anonymous and their non-committal to this project would not impact their existing relationship with the authors or Paralympics Australia in any shape or form (Evans et al., 2004; Pollard, 2015; Upasen, 2017). The *meaningful coherence* of this study was captured through the achievements of stated goals as we discerned the perspectives of participants on talent identification and development in the Paralympic context. The technique of *critical friend* was also utilized as authors engaged in discussions to ensure personal bias and perspectives did not compromise or dilute the relevant meanings generated through the themes (Smith and Sparkes, 2012; Burke, 2016).

### Procedure and Interview Guide

The interview guide consisted of a series of topics along with probe questions to elicit and navigate discussion (Patton, 2002). This guide was organized into three sections. The first captured coaches' development in Paralympic sport, observing their experiences as they progressed in their careers. The second section gathered information on what coaches deemed as indicators of talent during the identification process, a list of factors that can be developed to support athletes' career progression, and challenges pertaining to the identification and development of athletes. The final section was an open question that asked participants to design an ideal program without resource constraints (financial, staff, etc.). Closing questions allowed coaches to ask any specific questions or expand on items previously discussed.

### Data Analysis

Using the NVivo (NVivo qualitative analysis software; Version 12), the interview recordings were transcribed verbatim and reflexive thematic analysis guided the data exploration process (Braun and Clarke, 2019). Using the interview questions, significant thoughts and patterns were noted through the re-reading of transcripts. A set of codes were developed to formulate the data domain that guided authors in identifying and generating shared meanings across the participants' responses. Through collaborative and reflexive discussions, the authors continued to refine, organize, and merge meanings that generated six themes of “familial involvement,” “the role of impairment in talent identification and development,” “biopsychosocial: interaction of constraints,” “individualized approach,” “searching for athletes,” “funding the system” (refer to Table 1 for the theme breakdown).

**Table 1:** Theme breakdown.

**Theme**	**Examples**
Familial Involvement	Overprotectiveness leading to athlete dependency
	Family sporting background fostering an environment for the growth mindset and unstructured play at home
	Resources to support athletes' early careers (equipment, travel, transportation)
The Role of Impairment in Talent Identification and Development	Previous sporting experiences (key for athletes with later-onset impairment)
	Potential for athletes to be classified in unfavorable classifications
	Competitive pool domestically and internationally for each classification
Biopsychosocial: Interaction of Constraints	Stable constraints (anthropometrics, hand-eye coordination)
	Malleable constraints (current residency, occupation/education commitments)
	Interaction between constraints influences development across athletes' career span
Individualized Approach	Different impairments, abilities, and personalities
	Due to the wide range of differences, each athlete is coached differently (coaching method, training structure, travel needs, psychological support, etc.)
Searching for Athletes	Wide range of programs utilized to try to recruit athletes: talent search days, school programs, supporting rehabilitation and hospital staffs, and talent transfer
Funding the System	Limited funding impacts sustainability of recruitment programs, high turnover of staff, loss of intellectual property, a small pool of pathway athletes including the lack of female participants

## Results and Discussion

### Familial Involvement

Parental overprotectiveness was seen as a detriment to the athlete's ability to adapt and cope with sporting demands (e.g., commute to training, travel to camps and competition, the pressure to perform, and expectations of consistency). This sheltering by caregivers was seen by some as inhibiting their drive to take risks, which were identified as a fundamental component to success in Paralympic sport. As Alexandra noted:

Wrapped in cotton wool mentality from parents. I see the over-protective parents and what that creates. I've grown up in the community with those kids. One of them is practically blind and he can negotiate his way through the town through the school without any need for any help. And just his parents from day one said, “You're going to have to be on your own at some stage in your life, so let's start it now.” Whereas the other one's getting nowhere because parent is right there all the time. You have to be able to be independent.

Coaches have highlighted the importance of athletes' level of independence for successful development in the Paralympic context (Tawse et al., 2012). Parent/caregiver overprotectiveness can diminish opportunities for athletes to become independent (Johnson et al., (n.d.), p. 37), increase pressure on athletes, and contribute to athletes' loss of feelings of ownership in decision making which contributes to sports dropout (Witt and Dangi, 2018). However, there are scenarios (e.g., athletes with more severe impairments) where parental involvement was a necessity, as illustrated by James:

Some of our guys, their health needs are so complicated, you need the parents. So, you have these competing theories of how it should be [limit parental involvement], but there is the reality of the health needs. Because if we do not have that parent then we are going to be in danger of seriously hurting that person if we take them away.

On the one hand, participants did not appreciate overprotective parents; however, they were cognizant that support especially in the form of resources was vital, especially during the early years of athletes' careers and this has been well-documented in the Paralympic context (Johnson et al., (n.d.); Radtke and Doll-Tepper, 2014; Patatas et al., 2018; p. 43).

Therefore, during talent search days^3^ (see Dehghansai and Baker, 2020 for a detailed overview of a talent search day process), HP staff were conscious to observe family and caregivers' level of involvement, and therefore athletes' amount of independence (e.g., preparation for testing, maneuvering around the venue and between testing stations), as highlighted by James:

Whether the athlete comes in and they are pushing themselves in a chair, whether they have handles on the back of their chair or not because then they are relying on someone to push them. The level of independence is probably what we are looking at.

HP staff also preferred families (parents and siblings) with sporting backgrounds because the competitive and nurturing home environment fostered independence and a growth mindset, allowed for unstructured and informal sport experiences, and created a more effective line of communication between coaches and parents. Research findings in AB and Paralympic context suggests children with parents and siblings involved in sport are more likely to participate in sport (Hopwood et al., 2015; Papadopoulos et al., 2020) and parental support plays a key role in a person with an impairment's interest to participate and maintain long-term participation in physical activity and sports (Mactavish and Schleien, 2004; Dodd et al., 2009; Rowbotham et al., 2011; Buckley et al., 2020). Siblings have also been identified in AB literature as role models for work ethic and partners for unstructured play furthering athletes' development of technical and psychological skills (Côté, 1999; Weissensteiner et al., 2009; Phillips et al., 2010). While research in the Paralympic context is limited, coaches' perspectives of familial involvement here corroborate with the limited disability-specific literature and the conclusions drawn from AB literature.

### The Role of Impairment in Talent Identification and Development

HP staff were confident in their ability to create an environment for athletes to acquire a high-performance mindset and habits while refining their sport-specific technical and tactical skills, but only *if* athletes entered their sport at the early stages of their careers. Therefore, previous sporting experience was perceived as a vital component for athletes with late-onset impairments. This perception is also starting to be supported with empirical data, with recent studies examining the sporting experience of Paralympic sport athletes reporting a high incident of experience in AB sports prior to impairment-onset for those athletes with late-onset impairments (i.e., impairments acquired in early-adulthood or adulthood; Dehghansai et al., 2017b, 2020d,f; Dehghansai and Baker, 2020). Considering the quantitative nature of past work, authors were limited in drawing conclusions. They proposed the need for qualitative work to better understand the underlying reasons for a disproportionate number of athletes with later-onset impairments currently in the system with AB sporting experiences relative to those of the same cohort without experiences in AB sports. Findings from the current study suggest HP staff are actively looking for athletes with prior sporting experience, especially for athletes with later-acquired impairments.

Participants highlighted not only the perceived impact of early sport participation on achievements later in athletes' careers (i.e., acquiring fundamental movement skills, exposure to training and competition), but also, the positive by-products athletes acquire that are transferrable to other aspects of their lives such as independence, confidence, active involvement in the community, social skills, and sense of belonging. Sporting experiences play an integral role in shaping individuals' physical, psychological, and social well-being (Macdougall et al., 2015) and more importantly, it helps individuals strengthen their self-identity through sport (Allan et al., 2018). For some, early sport participation could be integral to ensure they are embedded into the sporting system while learning to master the abilities and limitations set by their impairments, as alluded to by Alexandra:

Once they hit 13, puberty, self-conscious issues, that is bad enough when you are just an [able-bodied athlete]. Throw in a disability aspect of it and they are not likely to engage in sport because they are already worried about being different and now, they have got that added difference because of their disability.

While athletes' impairments can play a major role in their involvement in sports, it is also integral to athlete identification, where HP staff consider (a) athletes' potential classifications, (b) current classification depth domestically and internationally, (c) their functionality/performance capacity based on their impairment, and (d) athlete needs and support due to their impairment. The role of “disability” was prominent in talent identification and development conversations, with Sandra echoing sentiments similar to the rest of the HP staff:

So, the first thing is their disability^4^. There tends to be a big range within the class, so someone like [athlete 1] in class [x], as compared to [athlete 2], in class [x]. [athlete 1] might be super, hardest trainer ever, committed, does everything, but his disability affects his chances to actually ever win a medal in that class. He would be more at the mid-to lower range of that class. That first and foremost is an inhibitor.

While HP staff preferred inclusivity and opportunities for all the athletes entering their system, they had to be methodical in how they distributed the very limited resources (i.e., lack of funding, limited time due to filling multiple roles, and having limited staff), which is an implication of the systemic issues discussed later. The importance of international success and limited resources to reach these expectations illustrates the strategic approach HP staff adopted to recruiting athletes based on impairment-related factors, as highlighted by Andrew:

We have tried to target the lower functioning athletes which is where the pools are smaller. Whereas [higher classes], it is very difficult just because of the numbers [i.e., more competitive]. If we found athletes in lower classes, we can make quick gains.

Thus, most athletes identified at the beginning stages of their careers were more likely to receive information pertaining to local clubs along with a list of expectations to work toward to demonstrate their potential. These expectations varied between sports with some focusing on performance benchmarks (e.g., Para cycling) and others on sport-specific skills (e.g., Para table-tennis, wheelchair rugby) which were most often assessed during regional and national tournaments.

### Biopsychosocial: Interaction of Constraints

HP staff highlighted key biopsychosocial constraints that can facilitate or inhibit athletes' development as they worked toward these expectations. First, participants identified psycho-behavioral factors as important for successful development including (a) resilience, (b) high work ethic, (c) response to pressure, (d) independence, (e) commitment to a high-performance lifestyle, and (f) a positive attitude toward previous challenges and anticipated upcoming barriers. Similar traits have been reported in the AB literature as indicators of high-performance success (Roberts et al., 2019). However, no specific testing tools were used, rather, HP staff relied on observation (“coach's eye”) during physical testing (for a–d), and informal discussions with athletes and their families post-testing during talent search days (e and f). As indicated by James:

Taking the time to talk to the families and the athletes during the day, getting a bit of an understanding about if they are spending their time complaining about not being able to get anywhere, nobody is helping them, nobody understands, [etc.]. You know, if they have already got a negative mentality about the system or whether they have got a mentality that [indicates resilience]. I think attitude is a huge factor.

During talent search days, HP staff objectively captured some limited physical attributes, including targeted anthropometric measures and hand-eye coordination tests. HP staff perceived these attributes to be stable and/or difficult to change over time and would not be worth the expansive resources (time and energy) required by the athlete and coaches to try to position the athlete for success in a specific sport. In their proposed model, Dehghansai et al. (2020a) refer to these factors as *stable structural* (e.g., anthropometric) and *stable functional* (e.g., hand-eye coordination) individual constraints which interact with other factors within athletes' environment across their development. Parallel with recommendations in this model, HP staff considered the interaction of these factors with other constraints in athletes' development and evaluated the disadvantaged position athletes may be in later in their careers competing among a more homogeneous group of athletes at the high-performance level. As Tom highlighted:

We got a guy, loves [the sport], he will even ask me questions, but he is not in the high-performance category. But he is always asking me stuff. How can I do this? Can I change something in my chair? Because he is so short, the poor guy sitting in the chair looks like a 10-year-old kid. He is never going to be a high-performance athlete, unfortunately.

Contrastingly, situational factors such as *interpersonal* environmental constraints also impact athlete development (see Dehghansai et al., 2020a), but are considered malleable and can evolve; HP staff highlighted that they considered these factors during the initial selection process. For instance, coaches considered the location of athletes' residences and whether the targeted sport can support the athlete in their current circumstances (i.e., access to training centers, equipment, and/or local coaches/clubs) in order to facilitate their development. Lack of resources (i.e., coaches, trained staff, programs) and inaccessible facilities is a longstanding issue pertaining to Paralympic (and other disability) sport (Radtke and Doll-Tepper, 2014; Martin Ginis et al., 2016; Patatas et al., 2018), and here, HP staff emphasized its direct impact on any further consideration and progression from the talent identification stage. A second barrier was athletes' ability to invest the appropriate time to become a high-performance athlete (e.g., extensive training, attending training camps, competitions abroad), which may require sacrifice in other areas of life (e.g., pausing or withdrawing from other educational/vocational developments, Schaal et al., 2011; Foskett and Longstaff, 2018). Dehghansai et al. (2020e) reported Paralympic sport athletes in their study often terminated their careers and education, with some moving cities and countries to focus on preparations for the Tokyo 2020 Games. As such, HP staff considered athletes' commitment to these other domains and potential interference with their sporting commitments. However, HP staff recognized the importance of approaching each situation individually with considerations to the interaction of constraints for both the immediate and long-term opportunities.

### Individualized Approach

HP staff outlined a wide range of factors that are considered for optimal development. First, impairment knowledge was a key component for coaches. Unfortunately, due to a lack of available impairment-related resources, coaches often lamented that they had to learn on their own, through trial and error and in-depth communication with parents and athletes (Radtke and Doll-Tepper, 2014; Lepage et al., 2020). Literature has previously highlighted that Paralympic coaches utilize different methods of learning (i.e., non-formal, informal) to combat the lack of resources and support available, particularly early in their careers (McMaster et al., 2012; Tawse et al., 2012; Turnnidge et al., 2012; Taylor et al., 2014; Lepage et al., 2020). Aligned with this, HP staff in this study specifically reported self-educating on classifications and impairment to better prepare for talent identification and development. There was also a notable reflection on the evolution of the classes and anticipating how classes may evolve in the future. Classification systems are a unique and evolving aspect of Paralympic sport that can facilitate or inhibit athletes' progression; coaches see it as an integral part of their role to understand the placement of an athlete within the system for athletes to succeed long-term (Radtke and Doll-Tepper, 2014; Patatas et al., 2020).

Dehghansai et al. (2020a) proposed a framework that considers a wide range of interconnected factors that contribute to the dynamic developmental environment. Highlighting the potential impact of this framework, insights from HP staff demonstrated the nuances associated with athletes' impairment, classifications, as well as secondary impairments and/or psychological disorders; thus, a truly individualized approach was considered for each athlete (Dehghansai et al., 2020a; Patatas et al., 2020). Impairment-related factors influence how athletes respond physiologically and whether they can execute a task based on their physical abilities. In addition, understanding athletes' prior experiences in and outside of sport helped coaches design more effective environments to reduce negative feedback and maximize engagement and positive experiences to ensure athletes approach training with the appropriate mindset. Louis summarized the complexity and the need for empathy to approach each athlete individually to fulfill their needs:

You get someone with a spinal cord injury, obviously they cannot take the heat as well so you just got to be very careful when you ask them to try something, and MS, you have got to be aware that they cannot do [certain tasks] because of their balance issues.

Impairment knowledge was not only important for sport-specific (e.g., classification, training response) purposes, but for other significant factors such as how athletes responded to travel. For example, Andrew shared a negative experience for them and their athlete which became a learning opportunity to prevent future mishaps:

The worst one was going to Fiji with the plane that did not have an aisle chair for one of the athletes. Having to carry her to the toilet was difficult for her, really difficult for her, to the point of her incurring an injury. That affected performance and her results at the event.

It was also important to understand the psychological impacts (e.g., anxiety and/or depression related to incident trauma) to better prepare for stressful environments that athletes are exposed to, and to develop contingency plans on how to deal with these circumstances. The high-performance environment can be very demanding, and the culmination of stressors, internal and external pressure can create an environment that is physically and psychologically draining (Fletcher and Scott, 2010; Lara-Bercial and Mallett, 2016). Frank explained the importance of understanding the unique psychological support needs for each athlete and the variability between athletes:

Knowing how to deal with athletes when they are uptight, and how to deal with nerves, [etc.]. It is not a one size fits all. Each athlete, you got to treat differently. Some you got to be really firm with. Others, if you are really firm with them, they will crack.

Therefore, there is not only a striking need to support coaches in better understanding impairment and providing sufficient resources on key physical indicators to optimize training environments, but the individuality of athletes' experiences also emphasizes the importance of supporting coaches on how to respond and support athletes' psychological needs.

### Searching for Athletes

HP staff shared a wide range of strategies utilized for athlete recruitment, whether it was working independently or partnering with state and national bodies (e.g., Paralympics Australia), These included talent search days, school programs, supporting rehabilitation and hospital staffs, and talent transfer from other high-performance Paralympic programs. During talent search days, it was seen as important to have experts with sport-specific knowledge. However, it was equally, if not more, important to have support from staff with an in-depth understanding of impairments and classification systems (Radtke and Doll-Tepper, 2014; Patatas et al., 2020), as highlighted by Alexandra:

Number one, we have got a sport who have coaches looking at athletes, but not understanding the impairment. We have also got sports who, work on a membership base so anybody who comes to that door, “We want them all to do [our sport]” Not so much, “This guy has the potential based on where he fits in the classification.” Or, “Maybe you should look at another sport.”

As such, HP staff with classifying experiences were able to help with the efficiency of selection during talent search days and appropriately set athlete expectations as alluded to by Alexandra:

Knowledge of classification is really helpful for [talent search days]. So [my colleague] and [my] classification knowledge is good, [so] we were able to say, “Look, I know you have written down here that you are interested in athletics, but the reality is you are on the low end of your class. Do it for fun, obviously. If you enjoy it, do it. But if you want to go through the pathway, here is what we recommend your best options are.

And HP staff not only took into consideration athletes' impairment and their abilities but also the depth of competition within that classification domestically and internationally.

Some sports have attempted to use coach development programs (aligned with their AB program) to educate local coaches on impairment-related factors and designing inclusive training programs that can be delivered at any club. As Kenny noted:

The coach development through the able-bodied pathway is very important for that. Developing all the coaches in Australia to know the basic of the sport. If someone [with an impairment] comes [to the club], they are not lost. They might not be full-on, but at least they are not lost, and they know who to contact if they want further information. They have got also a rough idea of the disability. If you have got a kid, a 7 year old in a chair, just put him with the other kids. That is okay. Another chair for her. Same. You do not do the ladder [exercise], you zigzag, whatever. That is inclusion.

Other attempts to support local clubs included additional equipment and resources to take on athletes with impairments.

Considering one of the common methods of “recruitment” as indicated by HP staff was “drop-in” (i.e., athletes initiating contact with a local club), supporting local clubs was a strategy to ensure stakeholders with the first point of contact were equipped with the necessary skills to support athletes' initial experiences (Radtke and Doll-Tepper, 2014; Patatas et al., 2018). Previously, sports also aimed to design programs to “upskill” and educate staff in industries that work with individuals with impairments including rehabilitation centers and school teachers. Research has shown rehabilitation centers play a key role in the introduction and integration of persons into sports post-injury (Wu and Williams, 2001; van der Ploeg et al., 2006; Radtke and Doll-Tepper, 2014; Patatas et al., 2018). Educating those in positions of “first contact” (e.g., medical staff in rehabilitation centers) was critical for ensuring patients with acquired impairment had knowledge of available resources to continue sporting activities upon leaving rehabilitation programs.

There were also approaches to recruiting athletes through informal “talent transfer” between sports, where an athlete may have the opportunity to advance further in a different sport. While informal and formal transfer programs have been investigated in the AB setting (e.g., Halson et al., 2006; Collins et al., 2014; MacNamara and Collins, 2015), there is limited research on the effectiveness of talent transfer programs in Paralympic contexts. In particular, it is unknown whether this type of approach can be formalized to maximize talent retention while ensuring athletes are supported through the transfer process. In summary, while HP staff attempted to utilize a range of strategies to identify potential athletes, the main challenge was finding sufficient funding to maintain the program and hire skilled staff.

### Funding the System

Participant insights suggested that limited funding was an overarching factor impacting athlete identification and development. Policy and limited funding have restricted sport organizations' capacity to maintain an effective pathway system with sufficient staff training (Radtke and Doll-Tepper, 2014; Patatas et al., 2020). In this study, participants shared similar experiences with funding limiting opportunities to support on-going recruitment initiatives (e.g., school and rehabilitation educational programs) or hiring of specific roles to increase initiatives which in turn reduced sport exposure within the community. HP staff also shared particular frustrations about the limited capacity to support pathway programs and to provide local coaches/venues with key resources (i.e., equipment, venue time) to ensure opportunities for athletes at the recreational level. Limited resources increased the demands of HP staff, forcing them to wear multiple hats and subsequently, increase their workload (Tawse et al., 2012). There were also concerns regarding how to support athletes once they entered the system, including the provision of equipment (i.e., lack of generic equipment to sample sports, costly equipment), coaching (i.e., lack of local coaches with impairment knowledge), and training (i.e., available facilities, training programs, cost of transportation to training camps).

Some of the participants alluded to a historically disjointed funding structure that supports both high-performance and recreational level sports as a particular challenge. HP staff understood the importance of the focus of the Australian Government to increase participation across the recreational level due to its benefits to the overall health of the individual and society at large.

From the sporting systems' perspective, most of the focus is on medal attainment with the majority of funding structured around the high-performance hubs and athletes. Therefore, the developmental pathway is lacking the necessary support from both streams to sustain the capacity to support athletes from recreational to high-performance, as Sandra further explained:

That is all about getting kids in schools playing sport more often. Then, for us, the challenge is then to transition them into clubs, then they've got the state, then you've got your national. It's always a vicious cycle. They want you to win medals, but to win medals you need a lot of money. A gold medal can be up to a million dollars investment in one athlete. We did some figures around how much for the Australian Winning Edge's model, the base amount of funding that a sport should be receiving, just to meet all those requirements, is half a million dollars. Most of us get a lot less than that. We have achieved a medal at the Paralympic level, at that time, with $290,000.

Many of the HP staff shared their concerns regarding the lack of depth at the lower levels and how this can be of concern looking ahead to the Paris 2024 Games and beyond. Edgar explained how the current structure and lack of funding for the pathway have put the pool of potential athletes for the Paris 2024 Games at risk:

I think that mainstreaming in terms of the national federations being responsible for the pathway has had challenges. I think the challenge is they have been provided funding to deliver a high-performance program for the Para perspective, but they have not really been provided the resources to deliver the pathway for Para athletes. So, I think there are plenty of gaps, there is a lot of work that needs to be done by the sports commission and national federations to really close some of those gaps. Because I think not only are we in jeopardy of not getting a great result in maybe not. Tokyo might be okay but 2024, I think we have got some real issues if our goal is to finish high on the medal tally at the Paralympic Games.

There are concerns regarding the impact of limited funding on recruiting, retaining, and developing athletes (e.g., quality coaching, opportunities for competition). With the weak pathway system, HP staff suggested an unhealthy consequence for athletes at a high-performance level, who potentially may have felt less pressure and had fewer concerns for internal competition for their spot leading to a culture of complacency. The limited pool of athletes among a small cohort of the population who have an impairment, coupled with budgetary constraints to expand resources and recruit more athletes puts HP staff at a clear disadvantage in trying to identify and develop athletes. James illustrated metaphorically how hard finding talent in the current system is:

We see a lot of kids over a whole year, a lot of kids. In some respects, [our sport] is an easy sport to deliver because it is so simple. There is that come and try level where it works really, really well, but to find the classifiable athletes and the ones who are going to take it seriously and can actually go from being a hit and giggle player, to a serious athlete, that seems to be so hard. You know the concept you are looking for a needle in the haystack? The problem is you are looking for a *golden* needle in the haystack.

This type of targeted approach to recruitment is even more difficult when looking for female athletes. Some sports see an obvious advantage of having a female on their roster as they receive additional classification points in international competitions, and many others acknowledged that female classes often do not have the same depth as their male counterparts; that said, many still have trouble recruiting female athletes. Recent studies have demonstrated the lower number of female participants in Paralympic sports including attendance in talent search days (Dehghansai and Baker, 2020; Dehghansai et al., 2020b). Even at the highest stage, there has been a consistent imbalance of numbers of female participants in comparison to male athletes at the Paralympic Games. While the recent Rio 2016 Summer Paralympic Games had a record high of 1,671 female participants, this was significantly lower than the male counterpart (*n* = 2,657) (Rio 2016 Paralympic Games, (n.d.)). HP staff in this study suggested puberty-related factors (e.g., self-consciousness of appearance) may play a role in female athletes' lower participation rates, especially if not introduced to sports prior to adolescence, as Edgar explained:

If someone has got a congenital impairment and they have not been captured in some way when they are at school age, then it is a bit of a challenge beyond that. And particularly for females with a disability if they have not been introduced to sport in some way, by the time they are about 13 when they care about their appearance a bit more, a bit more self-conscious, if they have not been captured at that age or around that age then it is pretty hard to get them back at 16 or 17 and beyond.

Recent work by Buckley et al. (2020) highlighted the family's role (i.e., being supportive and encouraging) in facilitating female athletes with visual impairments to participate in sports and develop an athletic identity. Here, Alexandra expressed how parents' overprotectiveness may play a role in a predominantly male environment:

A definite issue here is the lack of females in the system. I know it is an issue at 13–15. But I wonder if it is the over-protective nature of parents. You know, guys are going to be a little bit more out there. I do not know what the cultural reason is for that, but it could have an impact.

Literature examining the interaction of gender roles, puberty, and sport participation has highlighted the conflicting status between being an athlete and negotiating the role of femininity, and this crossroad is further complicated by the intersection of disability (Shakib, 2003). These environments position parents to be more “hands-on” in the decision-making process, and at times, minimizing unwelcoming environments to protect their children (Cirdland et al., 2014). On the other hand, there is literature suggesting sport participation for females prior to adolescence can address issues pertaining to self-consciousness and discontent that occurs during adolescence (Davison et al., 2007). Therefore, there is a need to develop initiatives to educate families on sport benefits and provide optimal environments for sport participation for young females with impairments.

In addition to systemic factors impacting the number and quality of athletes, limited funding also impacts the quality of staff and their experiences in Paralympic contexts. Due to budgetary constraints, most staff in the pathway are either volunteers or underpaid, with local clubs not having sufficient resources to educate staff on impairment-specific knowledge (Radtke and Doll-Tepper, 2014). These underpaid and unappreciated roles are usually met with high turnaround rates resulting in loss of intellectual property and reoccurring of the cycle with Edgar highlighting his frustration on these circumstances:

The big challenge for us is the turnover of staff and most people that work within [the organizations] do not grow up working in the disability industry. Some might work in sport but particularly when things like classification can be relatively complex in some sports and then to have staff that you work really closely with and they understand the network and the system and they are able to join lots of dots.

The limited funding is exacerbated in sports where equipment is an additional cost on top of already over-exceeding expenses (i.e., travel, accommodation, etc.). With technological advances, equipment is always evolving, and many are tailor-fit to the athletes, with limited opportunity to retrofit for someone else, therefore, each equipment is individualized and can play an integral role in how the athlete performs giving an advantage to athletes that can afford technological innovations. As alluded to by Wong (2008) and subsequent studies (i.e., Kean et al., 2017; Patatas et al., 2018), the rising cost of equipment can limit the growth potential for some athletes. On the other hand, technological innovations maximize the performance output for athletes who can afford these advances (Hambrick et al., 2015). James expressed their frustration on the competitiveness of equipment and the role technology plays:

Equipment is constantly evolving. If you are not innovating everyone else is. And to help, even if you have got the best coach in the world, your equipment has to be up to scratch. It is a bit like there is a bulls race going on. It is all about the bulls. And not just accepting that something half thought through is okay, that you got to be as fussy as maybe Niki Lauda is on his Formula One car. That you do not just accept what people are willing to do, you got to tell them actually what you need and make sure it is delivered.

In sum, systemic issues (policy, funding distribution, organizational communication) have resulted in limited funding opportunities across the pathway but have a marked impact on athlete identification and retainment. These funding limitations impact the quantity and quality of athletes, coaches, staff available in the system, but also limit opportunities to develop resources (coach education programs, relationships with community members, and organizations).

### Practical Implications

There is a range of practical implications emerging from the current study and what it means in the context of prior research. First, athletes' impairment and potential classifications are critical in the identification process. While, there has been criticisms of the medical approach to understanding athletes' impairments (i.e., focusing on the biological component of impairment rather than the disability construct), it is common for coaches to take this approach to frame their coaching strategies (Townsend et al., 2015). Therefore, ensuring coaches have an in-depth knowledge of impairment functionality and classification systems can contribute to more informed selection decisions (Radtke and Doll-Tepper, 2014; Patatas et al., 2020). However, coach education programs should consider the influence of social relationships that attribute to the impairment-related factors under evaluation (Townsend et al., 2015). As such, it is also important to consider the evolution of the Games and the classification system to ensure athletes selected today are positioned to benefit from changes to the classification system in the future. Second, knowledge of athletes and parents' sporting background, athletes' level of independence, and their behavior during search days was relevant in the identification process. This assessment occurs informally, alluding to the importance of conceptualizing, and formalizing this process to reduce biases in athlete identification. Research from the AB literature has demonstrated talent identifiers' implicit biases that influence their selection decision (Johnston and Baker, 2020). Systematizing and tracking selections based on objective measures may reduce chances of biases, but more importantly, will allow opportunities for reflection and correction to improve the identification process.

Within the Paralympic context, the impairment-related factors (i.e., classification, onset of impairment, experiences before acquiring impairment) suggest a need for reconceptualization of talent identification and development. In an ideal world, athlete development precedes identification, aiming to provide a rich environment for athletes to flourish and display their potential excellence. However, the classification component in the Paralympic contexts appear to impact this process by forcing HP staff to select athletes with impairments that align with functional specifications of their sports prior to developmental opportunities. This approach can have debilitative affects for persons that do not fit the classification criteria and/or are looking to sample sports recreationally; however, it does inform athletes of their potential in a given sport before they invest extensive training hours.

While it is difficult to make recommendations regarding how to increase funding for Paralympic sports, it is important to note the impact of limited pathway funding on the entire pathway, including the lack of qualified staff to recruit and retain athletes and loss of intellectual property due to unsustainable support models (Radtke and Doll-Tepper, 2014). This contributed to the limited athletes to develop for subsequent Paralympic Games, but then impacted through a lack of competition for national spots changing athlete behaviors (i.e., complacency).

HP staff highlighted the importance of sport opportunities at younger ages (i.e., pre-adolescence) to allow athletes to feel comfortable with their abilities and the sporting environment while developing fundamental skills. The current assumption on overprotectiveness of parents and especially the lack of opportunities for female athletes early on in their lives, appeared to be a contributing factor to the low number of females in Paralympic sport, highlighting the importance of creating female-specific initiatives to support their early entry and to create resources for educating parents on the benefits of sport participation for their children. Last, there was a complex interaction of factors that could impact athletes' development (Dehghansai et al., 2020a), and when identifying and developing athletes, HP staff considered these stable constraints in combination with more malleable constraints that evolve across athletes' careers. Some of these malleable constraints were directly tied to opportunity-cost (i.e., athletes' current residency, available resource to support remote athletes), therefore, there is a need to identify cost-effective initiatives (e.g., partnership with local venues for accessible resources) and maximize coaching opportunities (e.g., educational resources on impairment and sport-specific nuances for development coaches and volunteers, remote-coaching) to create a more sustainable environment to support athletes in remote areas with limited resources.

### Future Directions

For many of the participants, learning about the nuances of impairment (i.e., impairment type, classification) and associated implications (physiological response to training, psychological impacts of injury) was done informally due to limited available resources (McMaster et al., 2012; Lepage et al., 2020). While research in this area has increased recently, developing resources to support coaches can help ensure a safe and optimal environment is created for athletes, as well as inclusive coaching education programs for all mainstream (AB) sports to address a clear lack in recruitment and retention of athletes with impairments. As noted above, examining current sporting environments to better understand the lack of females in Paralympic sport remains a key area of further work. HP staff suggested the combination of puberty, disability identity, sporting environment, and familial role impacting females' interests in Paralympic sport. Focusing on extracting the factors that contribute to the disproportionate number of females in sport could be key to designing appropriate programs and initiatives to support female athletes' sport participation.

Two informal methods in the identification process, namely, the “coach's eye” and talent transfer initiatives, could be improved with formalization and empirical evidence is necessary to identify elements of their structure (Christensen, 2015; Jokuschies et al., 2017; Lund and Söderström, 2017; Romann et al., 2017; Roberts et al., 2019; Sieghartsleitner et al., 2019). Conceptualizing and systemizing coaches' approaches to assessment and selection could help track the identification process, refine and improve selection methods, and, more importantly, design programs to educate new coaches on strategies that experienced coaches use to select athletes. Similarly, formalizing talent transfer initiatives could help optimize sports' limited resources and ensure athletes are supported accordingly during and post-transition process.

### Limitations

While findings from this project contribute to a limited work in Paralympic talent identification and development, there are a couple of limitations for consideration. First, the reality explored here are specific to the Australian Paralympic system. While findings do align with the limited work from other nations (Radtke and Doll-Tepper, 2014; Patatas et al., 2018, 2020; Dehghansai and Baker, 2020; Dehghansai et al., 2020b,c,d), generalizations outside of this context should be done with caution. Second, it would be important to obtain the experiences of athletes within the system to identify developmental gaps from athletes' lens.

## Conclusions

There is very limited information on talent identification and development in Paralympic contexts. This study aimed to develop a better understanding of the Australia Paralympic talent identification and development through conversation with domain experts across a range of sports. It is clear that impairment and classification are pertinent factors to (initial) selection and therefore successful development (Radtke and Doll-Tepper, 2014; Patatas et al., 2020). However, there were other factors including familial involvement, biopsychosocial factors, and stable and malleable constraints that HP staff considered during the identification process. There were also long-standing issues (i.e., pathway funding, limited resources, and skilled staff) that appear to be consistent with literature from other nations (Radtke and Doll-Tepper, 2014; Patatas et al., 2020). Future research should examine how the current system can be designed to better track talent identification in a formalized method while creating resources to support coaches in the development of athletes and designing initiatives to introduce a more welcoming space for female athletes.

## Data Availability Statement

The datasets presented in this article are not readily available because providing transcriptions of the interviews will put participants' anonymity at risk. Requests to access the datasets should be directed to nimadehghan@gmail.com.

## Ethics Statement

The studies involving human participants were reviewed and approved by Research Ethics - Research & Innovation, York University. The patients/participants provided their written informed consent to participate in this study.

## Author Contributions

RP and ND were involved in the recruitment process, ND conducted the interviews and led the transcription and thematic analysis, all authors participated in the reflexive discussions to formulate the themes. ND structured the manuscript sections and RP and JB reviewed the manuscript and provided feedback. ND was responsible to prepare the manuscript for submission and ensured manuscript formatting aligned with the journal's guidelines. All authors contributed to the article and approved the submitted version.

## Conflict of Interest

The authors declare that the research was conducted in the absence of any commercial or financial relationships that could be construed as a potential conflict of interest.

## Footnotes

^1^In this paper, disability is used to refer to the biopsychosocial interaction of persons' biological impairment with their environment which creates the “dis” abled context. Impairment is used to refer to persons' biological conditions (International Paralympic Committee, 2014).

^2^Identification encapsulates the process of recognizing, selecting, and recruiting athletes into the system. In the event that one aspect of the process is more prominent to the content, the appropriate term is used.

^3^While it is more common in the able-bodied context to have participants of younger age attending these events, in the Paralympic context, participants of all ages attend the search days.

^4^The use of the term “disability” and “impairment” by interviewees were, at times, not reflective of our theoretical framework that is aligned to the International Paralympic Committee's mandate. However, we have kept the terminologies used in quotes directly as stated by the participants.
